# Advances and Pitfalls of Specialized Pain Care through Public and Private Health Care Providers in Catalonia and the Balearic Islands: A Physician's Survey

**DOI:** 10.1155/2022/4077139

**Published:** 2022-05-21

**Authors:** Javier Medel, Ancor Serrano, Carme Batet, Lluis Lorente, Susana Bella, Marta Ferrandiz, María-del-Mar Monerris, Sergi Boada, Jesus Villoria, Maria-Victoria Ribera, Antonio Montes, Sebastian Videla

**Affiliations:** ^1^Pain Unit, Hospital Universitari Vall d'Hebrón, Passeig de la Vall d'Hebron 119, Barcelona 08035, Spain; ^2^Pain Unit, Hospital Universitari de Bellvitge, Carrer de la Feixa Llarga, L'Hospitalet de Llobregat 08907, Spain; ^3^Pain Unit, Consorci Sanitari Integral, Hospital de Sant Joan Despí Moisès Broggi, Carrer Oriol Martorell 12, Sant Joan Despí 08907, Spain; ^4^Pain Unit, Institut de Medicina i Cirurgia de Barcelona, Carrer Bisbe Sivilla 46, Barcelona 08022, Spain; ^5^Pain Unit, Hospital Universitari Sant Joan de Reus, Avinguda Del Doctor Josep Laporte 2, Reus 43204, Spain; ^6^Pain Unit, Hospital Universitari de La Santa Creu i Sant Pau, Carrer de Sant Quintí 89, Barcelona 08041, Spain; ^7^Pain Unit, Hospital Universitari Germans Trias i Pujol, Badalona 08916, Spain; ^8^Pain Unit, Hospital Universitari Joan 23, Carrer Dr. Mallafrè Guasch 4, Tarragona 43005, Spain; ^9^Department of Design and Biometrics, Medicxact, Plaza Ermita 4, Alpedrete, Spain; ^10^Pain Unit, Hospital Del Mar, Passeig Marítim 25-29, Barcelona 08003, Spain; ^11^Department of Clinical Pharmacology, Hospital Universitari de Bellvitge, Carrer de La Feixa Llarga, L'Hospitalet de Llobregat 08907, Spain; ^12^Pharmacology Unit, Department of Pathology and Experimental Therapeutics, School of Medicine and Health Sciences, IDIBELL, University of Barcelona, Carrer de La Feixa Llarga, L'Hospitalet de Llobregat 08907, Spain

## Abstract

Optimal diagnosis and treatment of pain require a multidisciplinary approach that demands considerable coordination and forethought. A cross-sectional physician survey based on an online questionnaire was carried out to assess the adoption of multidisciplinary working patterns, compare the public and private models, and provide an update on the resources and organization of specialized pain care in Catalonia and the Balearic Islands. Active pain practitioners identified through the Catalan Health Service and Pain Society databases were sent an invitation in December 2020. Of the 321 physicians contacted, 91 (28.3%) answered and 71 provided complete responses (commonly anesthesiologists, representing 92 different sites; some worked at public and private sites). Up to 78.7% reported working in pain management teams, but only 53.5% were regularly involved in teaching or research activities. Thus, the proportion of multidisciplinary sites lies somewhere in-between. Median wait times were significantly shorter and within the recommended standards in private practices (e.g., 15 vs. 90 days in public practices for noncancer patients). In turn, private practices were slightly less staffed and equipped, albeit the differences did not reach statistical significance. Respondents made a median of 530 regular and 30 emergency visits per year, of which 190 involved interventional procedures. They offered a wide range of pharmacological and interventional therapies, although psychotherapy and the most sophisticated procedures were only available in ≤50% of sites. Pain clinicians and facilities are reasonably available in Catalonia, but barely more than half are truly multidisciplinary. Public and private practices differ in some aspects; the latter seems to be more accessible, but it is restricted to patients who can afford it. Compared to previous reports, this update shows both advances and outstanding issues. Multidisciplinary care could be expanded by incorporating more psychologists and some interventional procedures. The public practices should reduce wait times.

## 1. Introduction

Pain is among the leading causes of disability and disease burden worldwide, and its prevalence and impact are expected to grow because of population ageing [1, 2]. Although most of this burden is attributable to chronic pain, the adequate management of acute pain is vital to prevent chronification [3]. Pain prevalence is considerable. In Spain, surveys and cross-sectional evaluations found that about one third of adult individuals have experienced pain in the immediate past and one in five have suffered from chronic pain [4–6]. The figures are similar or even higher in other countries [7]. Significantly, up to one third of chronic pain sufferers may experience a severely disabling condition [8, 9]. Pain can be associated with considerable distress, psychological comorbidities, disability, and impaired quality of life [10, 11], or even premature mortality [12], and entails considerable costs from both direct healthcare expenditures and productivity losses due to work absences [13].

Nowadays, it is accepted that a broad, multidimensional perspective is required to understand pain and to treat patients adequately. The initial sensory input from disease-related neurophysiological changes elicits a range of cognitive, behavioral, and affective processes. These are further modulated by the patient's socioenvironmental context and eventually render and sustain the integral pain experience and its consequences [14, 15]. The understanding of pain as a multifaceted phenomenon has led to the development of sophisticated, comprehensive, interdisciplinary, and personalized therapies. These treatments are ideally applied at specialized centers, where a coordinated team can appropriately interact and work in an integrated facility that incorporates the necessary management services, equipment, and resources [16, 17]. Like in other places, national standards that regulate the operation of these pain treatment facilities have been established. Ideally they should encompass an heterogeneous, interdisciplinary team of healthcare providers and be able to manage both, complex challenging acute, with insufficient response to appropriate biomedical treatments, as well as chronic pain conditions in patients with demanding biopsychosocial needs [18]. Meeting such standards, nevertheless is challenging for many pain services [19, 20]. Meanwhile, undertreated pain continues to be a public health problem [21], despite its adequate management being considered a fundamental human right [22].

Pain treatment facilities should be periodically evaluated to address the level of compliance with the demanding standards, expose their organizational performance, identify difficulties and areas of intervention, and engage clinicians in reporting the quality of their care as part of quality improvement efforts [23, 24]. Survey research may well be a feasible framework to attain such goals that could otherwise be unattainable [25]. The Catalan Pain Society (https://www.scdolor.cat), committed to overseeing the quality of pain care, guiding the pain service provision in Catalonia, organizing local pain treatment structures and elaborating on the related healthcare processes, backed the present study to answer two relevant questions related to these tasks: To what extent have interdisciplinary or multidisciplinary working patterns been adopted by pain services, and what could be done to boost them? And, is there any major related difference between public and private models of care? The last question has been scarcely addressed in the past [23]. In turn, this research also offers a regional update of the resources, scope of services and activities of pain treatment facilities, and an assessment of their referral patterns, service utilization, process flows, and caseloads.

## 2. Materials and Methods

### 2.1. Study Design and Selection of Participants

This was a cross-sectional, prospective, multisite, questionnaire-based survey study. The target population comprised the whole of physicians of any medical specialty who dedicate their medical activity (total or partial) to managing patients with pain (active pain practitioners) in Catalonia or the Balearic Islands. A search strategy using several databases available to the investigators was designed and carried out (see the additional methods in Supplementary Materials). In total, 400 physicians providing care for pain were identified, of whom 321 were invited to participate and 91 (28.3%) provided answers (Figure S1). Sampling methods were not delineated because the initial intention was to obtain data from every member of the population. However, the low response rate led us to compare the reported characteristics with known external data on pain services (proportions of small vs. larger, public vs. private, standalone vs. hospital-based practices) to calibrate response bias [25], since response representativeness is more important than response rate [26].

### 2.2. Survey Instrument

This survey used an ad hoc questionnaire featuring 45 items (42 closed, 3 open) grouped into 5 sections that covered the participants' background, workloads and healthcare provision management, the resources available at their facilities, and the scope of the services provided. There is a complete description of this tool in Supplementary Materials. It was posted online through a validated remote data capture system and was active from January to March 2021. During this period, up to three reminder emails were sent to all selected physicians to stimulate participation. To compare both models of care, public and private, all items but those related to the participants' background were required in duplicate. The participants had to select the one applicable to their practice or fill in both if they provided care in the two settings.

### 2.3. Ethics and Transparency

The final study protocol was approved by the Clinical Research Ethics Committee of Bellvitge University Hospital in Barcelona (reference PR340/20). The need for written informed consent was waived. This study was carried out according to the stipulations of the Declaration of Helsinki and the level of protection of confidentiality concerning the protection of personal data as required by Spanish law (LOPD 3/2018). No remuneration was provided for participation.

The present manuscript was prepared in consideration of the preliminary checklist for reporting of survey studies (CROSS) [27].

### 2.4. Statistical Analysis

The proportion of truly multidisciplinary sites was estimated via the lower limit of the one-sided 95% exact binomial confidence interval (CI) of the sample proportion, which was calculated by combining the data on staff composition and the participation in management teams and teaching/research activities, features that distinguish the most complex and specialized types of pain facilities [18, 28–30]. Separate descriptions were done for the public and private healthcare settings to facilitate comparisons between them, which were performed using either Mann–Whitney U/Kruskal–Wallis tests or Chi-squared/generalized Fisher's exact tests, as appropriate. To adjust for multiplicity, the Type I error was contained by selecting contrasts that globally yielded a nominal false discovery rate (FDR) of 5% [31]. When the estimated number of true null hypotheses was greater than zero, we also calculated the adjusted *p* values (*q*-values) as the smallest estimated FDRs at which the test could be declared significant [32], constrained to be greater than or equal to the raw *p* values. Only compliant tests were regarded as significant. The sample size was not determined beforehand because the intention was to assess the entire target population. Nevertheless, given that participation was incomplete, we calculated that the precision (half-width) of two-sided 95% CIs for the observed proportions was 8.95% or higher when a sample of 92 (see Supplementary Materials) was drawn from a finite population of 400 individuals.

## 3. Results

### 3.1. Respondents' Background and Proportion of Multidisciplinary Sites

**Figure 1:**
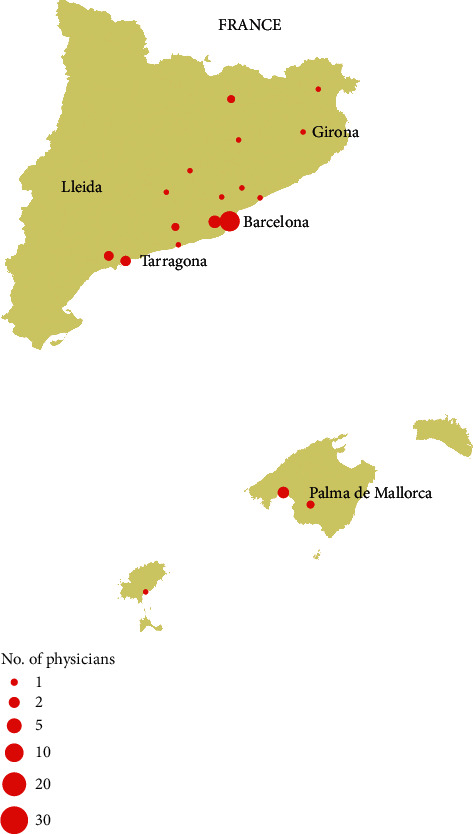
Display of the number of surveyed physicians per municipality.

The participants, commonly anesthesiologists with considerable clinical experience (Table 1 and Supplementary Materials), represented 92 different sites, 63 public and 29 private sites, and were distributed throughout 18 different towns in a dispersed geographical area (Figure 1). In many instances (77/89, 86.5%), they declared to be working in a pain center or clinic, and 70/89 sites (78.7%) were integrated into pain management teams; these proportions were somewhat lower at private sites (data not shown). Thirty-eight of the 71 respondents (53.5%) participated in research and teaching activities. Nearly all made contributions to conventions; the number of these contributions was significantly higher among physicians working in public and private practices (national conferences) or among those working only in the private setting (international conferences) (Table 1). About one-half contributed publications to biomedical journals and participated in research studies; the number of contributions was significantly higher among physicians working in both the public sector and private practices. Many respondents received rotating/resident physicians, and about two-thirds imparted training activities, mainly hospital general sessions, sessions to primary care physicians, and classes in universities. Compliance with standards was moderate (between 50% and 75% of sites, Table 1). The medical practice was mainly based on scientific evidence and clinical guidelines or protocols; personal expertise and experience were less cited.

Considering these data, the proportion of sites that can be regarded as multidisciplinary lies somewhere between 38/71 (53.5%) and 70/89 (78.7%), the proportions of sites in which respondents participated in teaching and research activities and being integrated into pain management teams, respectively. Thus, it can be confidently said that the true proportion of the target population is at least 43.1% (the lower limit of the CI of the lowest of these proportions).

### 3.2. Caseloads and Service Provision

Respondents performed a median of 150 first-time and 380 follow-up visits per year, of which 90 and 100, respectively, featured an interventional procedure. Second opinion visits were rare, yet significantly more frequent at private than public sites (Table 2). Median wait times were short at private sites (4.5 and 15 days for initial visits and 7 and 15 days for follow-up visits in cancer and noncancer patients, respectively) and long at public sites (7 and 90 days for initial visits, and 15 and 90 days for follow-up visits in cancer and noncancer patients, respectively); this difference was statistically significant for all types of visits (Table 2). Compliance with wait time standards was good in private sites and poor in public sites. In both settings, preference was given to cancer patients. Patients were often referred to pain facilities by trauma surgeons, primary care physicians, or neurosurgeons. About one-fifth of patients went on their own initiative, this proportion being significantly higher in the private setting. The main reasons for referral were poor pain control (especially in public settings) and the lack of a satisfactory pain diagnosis (more common in private practices) (Table 2). The need for interdisciplinary care and a better management of drug toxicities were also common. Supplementary Materials provide some additional results on service provision.

### 3.3. Resources and Staff

Information on facilities and services was available from 82/92 and 79/92 sites (89.1% and 85.9%), respectively. These results are summarized on Table 3 and detailed in Supplementary Materials.

**Figure 2:**
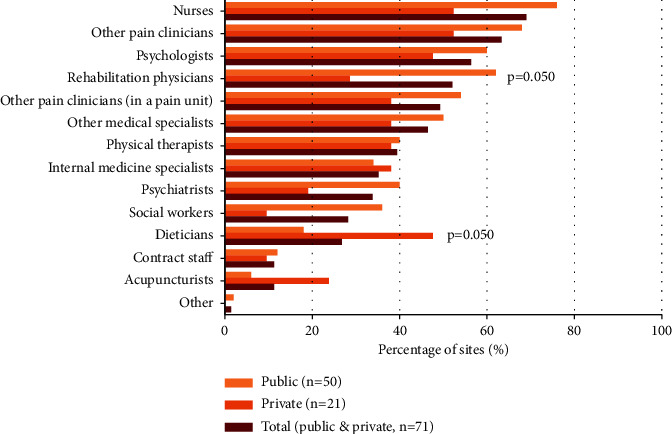
Availability of peer staff at surveyed sites. Multiplicity-adjusted *p* values (from Chi-square or Fisher's exact test) are indicated when there is a statistically significant difference between the groups (public and private).

Figure 2 describes the staff composition (other than the respondent) of the pain treatment facilities. Professionals who commonly worked with the respondents were nurses, who were present in 49/71 sites (69.0%), other pain clinicians in 45/71 (63.4%), psychologists in 40/71 (56.3%), and rehabilitation specialists in 37/71 (52.1%) sites. Nurses, social workers, and rehabilitation physicians were more frequent at public sites, although statistical significance was just reached for the latter after multiplicity adjustment, whilst dieticians were significantly more frequent at private sites. In 38/72 sites (52.8%; 50.0% public and 59.1% private), the respondents felt that the staff was insufficient. The professionals in demand in 70–90% of sites were physical therapists, psychologists, rehabilitation physicians, and nurses (detailed results not shown). Also, in private sites (14/16, 87.5%), the need for more pain clinicians was cited.

## 4. Discussion

This research has updated the general picture of healthcare management and resources for specialized treatment of pain in Catalonia and the Balearic Islands. Pain clinicians and services were reasonably available, although a substantial proportion did not offer multidisciplinary care and fell under national standards in terms of facilities and, at public sites, wait times. Some resources were scarcer in private settings, but these were more accessible than the public facilities and sometimes used as a second option, as suggested by the shorter wait times and the greater volume of visits scheduled directly by the patients themselves or appointments for a second medical opinion.

Like in a recent national report [33], just over half the respondents participated in teaching and research activities, but the estimated proportion of sites that could be regarded as multidisciplinary (over 53.5%) was slightly higher than nationwide [34]. Although the lower confidence limit of 41.3% is not above the national figure, the actual proportion is probably higher because multidisciplinarity does not necessarily entail teaching and researching [28, 29]. Figures in other countries were lower or similar, supporting the external validity of our result, yet the authors also faced difficulties in establishing the actual proportion of multidisciplinary facilities [20, 23, 35–37].

The lengthy wait times suggest enduring difficulties in accessing pain services. Except for the first visits of cancer patients, wait times at public sites were longer than recommended [38], even though they were shorter than in previous surveys [23]. As others have reported [23, 39, 40], private sites managed to operate with significantly shorter times and stay within the proposed ranges. Although our delays were in general well below the recommended 6-month maximum term, this should be improved to ease access for those who do not have access to private healthcare and avoid the deleterious effects associated with delayed pain care [41]. The latter is particularly relevant in patients referred to specialized care, who usually present emotional distress or psychiatric comorbidities as a result of a lengthy experience with pain [42] and who appear able to benefit from integrated psychological therapies [43].

A variety of healthcare workers should staff multidisciplinary pain centers or clinics including physicians, nurses, mental health professionals, and physical therapists [18, 28]. Notably, the availability of psychologists, psychiatrists, rehabilitation physicians, internal medicine specialists, physical therapists, and social workers was greater than at the national level [33] and compatible with our estimations of the availability of multidisciplinary services. However, psychologists, psychiatrists, and physical therapists, who are part of the required core staff [28, 29], were still lacking in about one-half of the sites. Although the national standards also allow for less developed structures regarded as “unidisciplinary facilities” [18, 30], they should at least have one psychologist [18]. Indeed, the need for more psychologists was frequently cited in this survey, and just one-fourth of patients received a psychological assessment during the initial visits. Paying more attention to psychosocial factors was already recommended one decade ago [44], since failure to address the psychosocial components of the pain experience can adversely affect the severity of symptoms, patients' adaptation, and response to treatment [44, 45], but there appears to have been little progress since then. Surveys in other countries have also reported a relative lack of psychologists [20, 23].

The initial search for practitioners providing care for patients in pain yielded a total of 400 for a population of 8,889,422 [46], which translates into a ratio of 4.5 per 100,000 people, within the range of other medical specialties in Spain [47]. Therefore, assuming that the prevalence of any type of pain and chronic pain is 33% and 20%, respectively (see Introduction), the resulting ratios are 7,334 and 4,445 patients per physician. These figures clearly improve previous reports, but they still do not match the availability of specialists for other chronic conditions [44]. Given the significant negative impact of long-term pain, it should be considered why chronic pain is not being prioritized as other long-term conditions [48, 49]. Besides the important personal and societal costs, improper specialized management of pain can also increase the burden of care [50].

Also, this improvement should be interpreted with caution because our procedure to identify pain clinicians may have been overinclusive, which cannot be verified without the information from those who failed to answer the survey. Furthermore, selection bias (which happens when respondents disproportionately possess traits that affect the outcome, such as much interest, longer clinical experience, or being more frequently employed at large-pain centers or clinics) is probable, since they are not a random selection of all physicians invited. In fact, the proportion of pain centers and clinics identified (77 sites, which would represent 42.1% of the total census of 183 units formerly compiled in Spain [33, 51]) clearly surpasses the 18.8% population share of the regions evaluated. Thus, even realizing that Catalonia has more pain facilities than the rest of Spain [52], nonresponders would probably work in less structured and equipped facilities than responders, who in some instances would also have regarded any organized specialized pain practice as a pain center or clinic. Nonetheless, even if only 50–66% of the surveyed sites were truly multidisciplinary (see above), access to such care would be approximately one per 174,000–228,000 people, in line with or above other countries [23] and Spain as a whole [34].

Facilities and equipment differed between sites. Some lacked certain resources required by national standards [18] and international recommendations [28]. Compared to previous surveys in Spain that revealed a number of issues, including the insufficient coordination with primary health care, the lack of designated spaces, inappropriate support staff, and the part-time dedication of practitioners [51, 53, 54], there was both improvement and stagnation. The facilities outperformed previous surveys regarding the availability of designated spaces, consultation/examination rooms, waiting areas, block rooms, operating theatres, and dedicated day hospitals. Day hospitals were in fact more widespread than in other regions of Spain [33] and other European countries some years ago [23, 55, 56]. On the other hand, especially at private sites, there was no progress in aspects such as the absence of nursing stations, administrative and staff areas, rooms for clinic sessions, and dedicated hospitalization areas. Although most facilities had their own space, it was generally deemed insufficient. Operating theatres were well equipped but typically shared with other hospitals or healthcare services.

A wide offer of therapies, including pharmaceutical and interventional techniques, was reported, although the most sophisticated interventions were available in half or less of the sites (see Supplementary Materials). The respondents suggested that this could be improved. Thus, the incorporation of either psychologists or interventional techniques could contribute to expanding multidisciplinary care; the cost-effectiveness of these two strategies should be assessed to set development priorities.

Setting realistic expectations with pain patients is crucial [16]. Remarkably, patients' expectations appeared to be appropriate as up to 50% only awaited relief instead of cure. In consonance with the biopsychosocial model [57, 58], practitioners should acknowledge this fact to avoid merely focusing on achieving cures, as this can become a source of frustration [59]. An opportunity is also provided to modulate the individual patient's expectations according to the potential benefits and disadvantages of a treatment, which could greatly impact patients' satisfaction [60]. Psychosocial interventions may also have an important role here: when cure is not possible, which is unfortunately very common, the success of biomedical interventions will greatly rely on how a patient is able to adapt and self-manage their symptoms [42].

The main limitation of this research stems from the low response rate. Nevertheless, our 28.3% rate falls within the expected participation in web-based physician surveys [61], which has been as low as 11% in other recent anesthetists' [62] or pain physicians' surveys [63]. Moreover, low response rates do not usually affect the internal validity, but representativeness [64]. However, we have already discussed how selection and response biases can affect the external validity of the results and have given consistent cautious interpretations. In this vein, as the responders most likely worked in more structured and equipped facilities than the physicians who did not respond (see above), the availability of services and resources may have been overestimated. However, the correspondence between ours and others' results in several aspects such as the availability of multidisciplinary facilities, the public-private difference in wait times, the lack of psychologists, or the size of population coverages supports the external validity of this survey. Indeed, we think that the participation of more than one physician per center is unlikely. Therefore, the proportion of centers represented would be considerably higher than that of physicians, as their geographical dispersion suggests (Figure 1). Validity apart, this fact boosts the study's reliability too, because it makes replication of the main results likely regardless of the particular clinician reporting from each site. Nevertheless, we could not quantify the actual number of participants per site because personal data protection laws prevented us from knowing their identities. Hence, the magnitude of bias attenuation is uncertain and as is some degree of data clustering that could not be accounted for in statistical inferences. Although we sought the views of several specialties, nearly all the respondents were anesthesiologists. Still, these are well placed to report on pain services, which they typically lead and staffed. Another limitation is that survey-based self-reported studies are subjected to reporting biases if, for instance, respondents tend to select more desirable outcomes. But as mentioned, the usefulness of physician surveys to evaluate pain treatment facilities is widely accepted [25]. Unobserved variables, such as the time since founding, the actual number of professionals, or the most prevalent diagnoses at each site, could partially explain why some standards were not met. Without this information, we could have overlooked potential intervention targets. Lastly, we have regarded the facilities staffed by diverse healthcare professionals as just multidisciplinary since the proportion of truly interdisciplinary centers, in which professionals share therapeutic aims and communicate regularly with each other to align on diagnosis, therapeutic plans, and objectives, is uncertain.

## 5. Conclusions

This regional update shows that multidisciplinary working patterns have not yet been universally adopted despite some advances in terms of accessibility, resources, and activities compared to previous reports. The private model of care seems to be more accessible than the public system just at the expense of a slight reduction in resource availability, and of nursing staff in particular, but it is restricted to patients who can afford it. Shortening wait times in the public system should be a priority to avoid delaying pain care for the numerous patients who cannot pay out of pocket for medical services. While some interventional techniques are still lacking in several sites, the shortage of psychologists and physiotherapists is of relevance. Future research should address the comparative cost-effectiveness between incorporating mental health professionals and the expansion of material resources and equipment, especially for the more sophisticated procedures.

## Figures and Tables

**Table 1:** Respondents' general characteristics and participation in teaching or research activities.

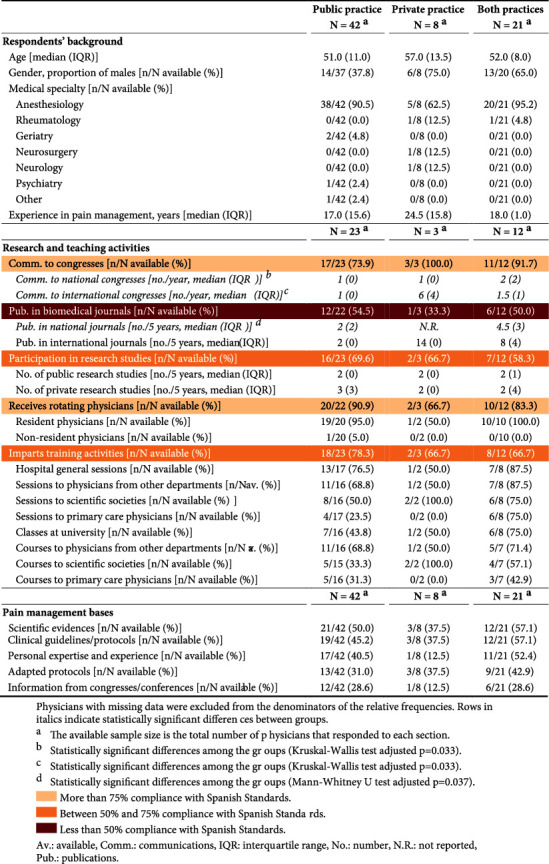

**Table 2:** Caseloads and patterns of service utilization.

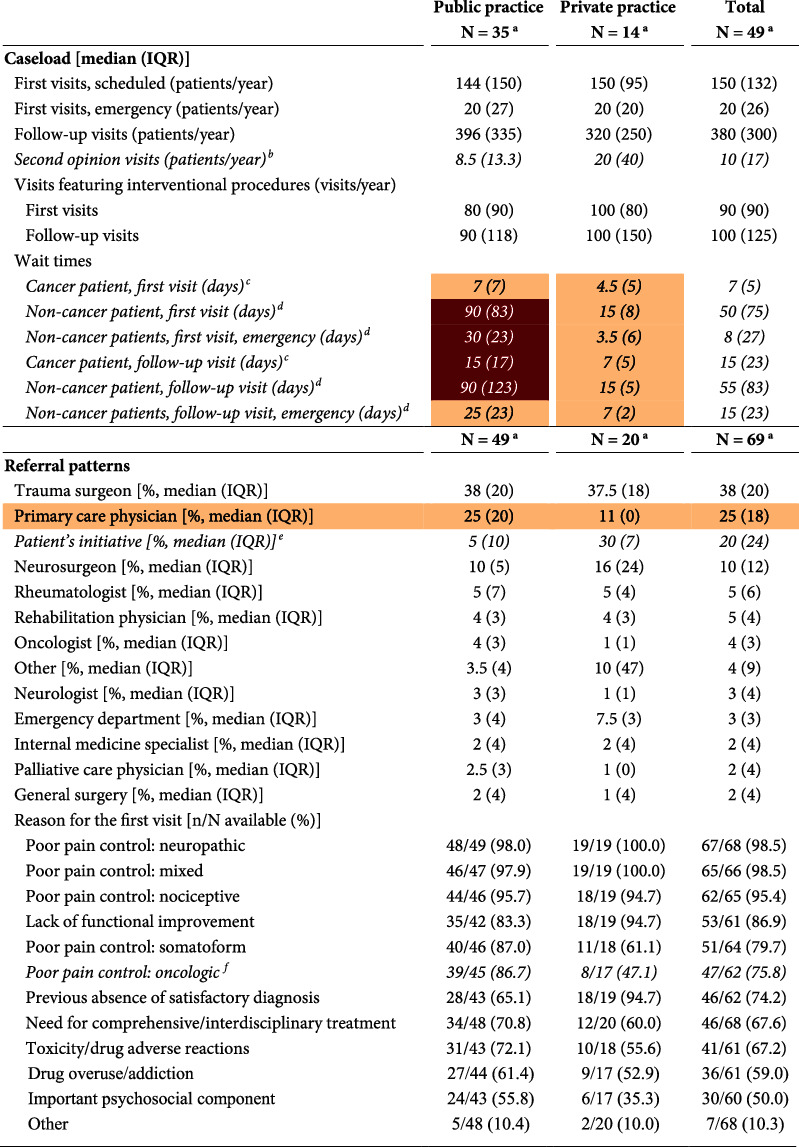
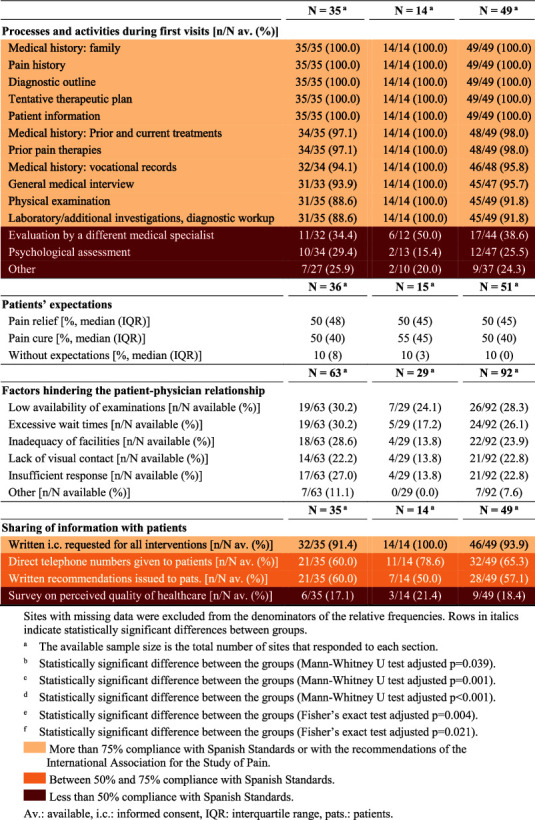

**Table 3:** Resources available at surveyed sites.

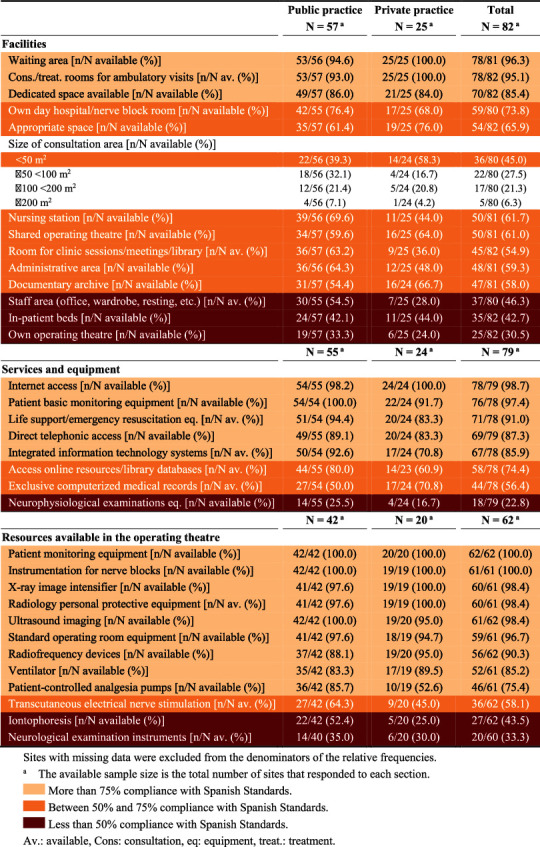

## Data Availability

No public access to the dataset is planned at this moment. Dr. Cristian Tebé, the Head of the Biostatistics Unit-IDIBELL, will oversee the dataset. Granting access to this information will be evaluated on a case-by-case basis, upon reasonable request by the interested party, and with permission from Dr. Antonio Montes, President of the Catalan Pain Society.

## Supplementary Materials

Supplementary Materials The file “CATDOLMAP v7-0 (Supplementary Materials)” contains the additional methods and results referred to in the main text.
