# Supplementary material for: Advances and Pitfalls of Specialized Pain Care through Public and Private Health Care Providers in Catalonia and the Balearic Islands: A Physician's Survey
Source: Pain Res Manag. 2022 May 21;2022:4077139. doi: 10.1155/2022/4077139 (PMC9148249; doi:10.1155/2022/4077139)
Supplement: Supplementary Materials — The file “CATDOLMAP v7-0 (Supplementary Materials)” contains the additional methods and results referred to in the main text. [file 4077139.f1.docx]

# Pain Research and Management

# Advances and pitfalls of specialized pain care through public and private health care providers in Catalonia and the Balearic Islands: A physician’s survey

**Author Information**

Javier Medel,^1^* Ancor Serrano,^2^* Carme Batet,^3^* Lluis Lorente,^4^ Susana Bella,^5^ Marta Ferrandiz,^6^ María‑del‑Mar Monerris,^7^ Sergi Boada,^8^ Jesus Villoria,^9^ Maria‑Victoria Ribera,^1†^ Antonio Montes,^10†^ Sebastian Videla^11,12†^

1 Pain Unit, Hospital Universitari Vall d'Hebrón, Passeig de la Vall d'Hebron 119, 08035 Barcelona, Spain. Email: fjmedel@vhebron.net

2 Pain Unit, Hospital Universitari de Bellvitge, Carrer de la Feixa Llarga s/n, 08907 L'Hospitalet de Llobregat, Spain. Email: a.serrano@bellvitgehospital.cat

3 Pain Unit, Consorci Sanitari Integral, Hospital de Sant Joan Despí Moisès Broggi, Carrer Oriol Martorell 12, 08970 Sant Joan Despí, Spain. Email: cbatetg@gmail.com

4 Pain Unit, Institut de Medicina i Cirurgia de Barcelona, Carrer Bisbe Sivilla 46, 08022 Barcelona, Spain. Email: llorcapa@gmail.com

5 Pain Unit, Hospital Universitari Sant Joan de Reus, Avinguda del Doctor Josep Laporte 2, 43204 Reus, Spain. Email: sbella@netics.cat

6 Pain Unit, Hospital Universitari de la Santa Creu i Sant Pau, Carrer de Sant Quintí 89, 08041 Barcelona, Spain. Email: mferrandizmach@gmail.com

7 Pain Unit, Hospital Universitari Germans Trias i Pujol, 08916 Badalona, Spain. Email: mmmonerris.germanstrias@gencat.cat

8 Pain Unit, Hospital Universitari Joan 23, Carrer Dr. Mallafrè Guasch 4, 43005 Tarragona, Spain. Email: sergib@mac.com

9 Department of Design and Biometrics, Medicxact, Plaza Ermita 4, Alpedrete, Spain. Email: villoriajesus@medicxact.es

10 Pain Unit, Hospital del Mar, Passeig Marítim 25-29, 08003 Barcelona, Spain. Email: AMontes@parcdesalutmar.cat

11 Department of Clinical Pharmacology, Hospital Universitari de Bellvitge, Carrer de la Feixa Llarga, s/n, 08907 L’Hospitalet de Llobregat, Spain. Email: svidela@bellvitgehospital.cat

12 Pharmacology Unit, Department of Pathology and Experimental Therapeutics, School of Medicine and Health Sciences, IDIBELL, University of Barcelona, Carrer de la Feixa Llarga, s/n, 08907 L’Hospitalet de Llobregat, Spain. Email: svidela@bellvitgehospital.cat

* These authors contributed equally

† Joint senior authors.

# Supplementary Materials

# Materials and Methods

## Sampling

Since the target population of this survey was finite (i.e., physicians of any medical specialty who dedicate their medical activity to managing patients with pain in Catalonia or the Balearic Islands), it was attempted to obtain data from as much members as possible. Public databases of the Catalan Heath Service and the membership databases of the Catalan Pain Society and the Academy of Medical Sciences of Catalonia and the Balearic Islands were searched to identify pain treatment facilities as well as specialized pain care clinicians treating patients because of their pain either full or part time. The Catalan Health Service databases include an exhaustive register of all licensed healthcare facilities operating in Catalonia, from large public hospitals to small private practices, featuring information about the range of healthcare services provided. All database records that contained the string “pain” in the services field were retrieved. The membership databases of the Catalan Pain Society and the Academy of Medical Sciences contain both updated personal contact data and information about the professional affiliations of each member. These affiliations were compared with the facilities selected in the previous step to identify all physicians working in any of the practices providing care for pain. For some practices, no physician could be singled out. In these cases, phone calls were made using the information provided in the database to reach the practitioners of the pain section, explain the nature of the study and collect their email addresses. A total of 400 candidate physicians were identified using these steps. A formal invitation email on behalf of the head of the Catalan Pain Society providing detailed information about the study and a link to the online questionnaire was then distributed by an assistant from the Academy of Medical Sciences in December 2020. For administrative reasons, the invitation could not be sent to 79 physicians who were not former members of the Academy; thus, a total of 321 invitations were finally delivered.

## Survey questionnaire

The investigative team developed the data capture tool based on those used in previous survey research [1-4] and the national standards for pain treatment facilities [5,6] as a checklist to examine it, balancing comprehensiveness and brevity to ensure content validity. This questionnaire (Table S1) featured 45 items grouped into 5 sections, of which 42 had just closed response choices. The 5 sections collected information about a) the respondent’s background, tasks and participation in teaching and research activities, b) the routine workflow, caseload and wait times at their facilities, c) the patterns and management of the provision of services, d) the site’s space, equipment, staff composition and resources, and e) the treatments and procedures offered. All items but 8 (that addressed some general information about the respondents’ backgrounds and their research/teaching activities) were required in duplicate, one for each model of care, public or private. There were 2 open-ended questions regarding the aspects most valued by patients that were not analyzed for this report.

The data were collected and managed using REDCap electronic data capture tools [7] hosted at the Bellvitge Biomedical Research Institute, which was responsible for the data management tasks. The system requested some personal data from participants before they were granted full access to the survey, so that they would only be able to fill it out once. At the request of the Ethics Committee, this personal data was neither available for the data analysts, the study coordinators nor any other researcher involved in the study. The survey was accessible until the end of March 2021. During the period it was active, up to three reminder emails were sent to all selected physicians to stimulate participation.

To better represent the trends in healthcare service provision over recent years, respondents were asked to focus on their experience and activity during the year 2019 rather than the immediate past, to avoid distortion caused by the coronavirus pandemic [8].

## Statistical Analysis

Means, standard deviations, medians, interquartile ranges (IQR) and percentages were calculated for continuous and categorical variables, respectively. Missing data were excluded from the analyses (e.g., results are provided only for the number of respondents/sites available in each item and percentages are relative to these numbers). Some physicians were counted twice in the analyses because they contributed responses for both settings, public and private. Thus, the units of analyses were the sites (92 in total) for all items but those 8 that were not solicited in duplicate (see above the description of the questionnaire), which were analysed for the physicians who completed the survey.

All analyses were done with the R environment (R Foundation for Statistical Computing, Vienna, Austria; available at: www.R-project.org) version 4.1.0. The map of the spatial distribution and number of participating physicians was drawn using the “plotly” package, version 4.9.4.1.

# Results

## Disposition and background of participants

Ninety-one/321 invited physicians (28.3%) responded to the survey, although 20 only provided partial responses (Figure S1). Forty-two/71 respondents (59.2%) were working in a public practice, 8/71 (11.3%) in a private practice and 21/71 (29.6%) worked in both practices; thus, responses were obtained from 92 different sites, 63 public and 29 private. The respondents’ median (IQR) age was 52.0 (10.6) years and 33/65 (50.8%) were males, being this proportion lower in the public sector (Table 1). Most (63/71, 88.7%) were anesthesiologists. They have a median (IQR) of 19.0 (13.0) years of experience in pain management. Sixty-one/63 (96.8%) public and 26/29 (89.7%) private practices were integrated into hospitals, in many cases within the Anesthesia and Reanimation departments (36/55 sites that responded, 65.5%, significantly more in public than private practices: adjusted p=0.001). Only 2 and 3 physicians in the public and private sectors, respectively, provided care at a primary center or a single private practice. Of all sites surveyed, 83/92 (90.2%) managed chronic and 28/92 (30.4%) acute pain conditions; 46/92 (50.0%) treated nociceptive, 56/92 (60.9%) neuropathic and 51/92 (55.4%) mixed pain types. These proportions were similar in public and private sites. Just 11/65 responding sites (16.9%) had certified their management system according to ISO standards. Barely half (35/64 responding sites, 54.7%) had a formal setup with other colleagues or departments to coordinate patients’ care, mostly involving cross-consultations (33/64, 51.6%) and pre-specified criteria to avoid unnecessary referrals (26/64, 40.6%). The latter arrangement was significantly more common in public (23/46, 50.0%) as compared to private settings (3/18, 16.7%) (adjusted p=0.044).

Figure S1: Flow diagram of study participation.

## Service provision patterns

Initial visits were quite comprehensive, typically including complete history taking, physical examination, diagnostic workup and therapeutical planning. However, the participation of peer staff other than the pain clinician, such as internal medicine specialists or psychologists, was scarce (Table 2 in the Main Document). Complete clinical reports were frequently issued, mainly at the patient’s request, covering medical history and physical examination, the results from the diagnostic workup, proposed diagnoses and therapeutic plans.

Patients’ expectations at the initial visits were evenly split (50/50%) between pain relief and pain cure. Physicians reported a low prevalence (7‑26/92, 7.6‑28.3%) of factors that could hinder their relationship with patients. Such factors were less common in the private setting, but the differences between groups did not reach statistical significance (Table 2 in the Main Document). Additionally, about two‑thirds (61/92) acknowledged the importance of empathy and good communication with patients for the success of their practices. At discharge, 32/49 (65.3%) sites provided patients with direct telephone numbers in case of emergency and 28/49 (57.1%) supplied written recommendations. Only 9/49 (18.4%) sites surveyed patients about their perceived quality of healthcare. There were no significant differences between public and private settings.

## Resources and services

Most facilities had a dedicated space to manage pain patients, but it was generally small (in 58/80 sites, 72.5%, the physicians had less than 100 m^2^ of space for their activities) and up to one-third of respondents (28/82, 34.1%) deemed it insufficient. Nearly all sites had waiting areas for patients, desks for outpatient consultations and included operating theatres (although 50/75, 66.7%, were shared), and about three-quarters (59/80, 73.8%) featured a day hospital/block room. Less frequently (47‑50/81, ~60%, Table 3 in the Main Document), the sites incorporated nursing stations, meeting rooms, administrative areas or documentary archives. In‑patient beds were available in just 35/82 sites (42.7%). Nursing stations, meeting rooms and staff areas were more frequent in public than in private sites, yet the differences did not reach statistical significance after multiplicity adjustment. Compliance with standards was in general moderate (Table 3 in the Main Document). Monitoring and respiratory support/resuscitation equipment was available in almost all sites. Even though information technology systems were adequate in most sites, only 44/78 sites (56.4%) had dedicated computerized medical records. All or nearly all operating rooms had monitoring equipment, appropriate instrumentation for nerve blocks, X-ray image intensifiers, radiology personal protective equipment and ultrasound devices (Table 3 in the Main Document). Also common were radiofrequency devices and ventilators. Less frequent were pumps for patient-controlled analgesia (PCA), transcutaneous electrical nerve stimulation or appliances for iontophoresis and neurophysiological examinations. Compliance with standards was in general good but for the later equipment, which was available in less than half of sites. PCA pumps and iontophoresis devices were less frequent in private facilities but, again, the differences did not reach statistical significance after multiplicity adjustment. Asked about the resources they would like to include to enhance their clinical practice, respondents frequently (23/32‑19/24, 71.9‑79.2%, detailed data not shown) mentioned neurological/neurophysiological examination equipment (for example, quantitative sensory testing devices) and exclusive computerized medical records. Of note is that 12/15 (80.0%) of the private sites mentioned the need of more nursing staff.

Information on therapies offered is available from 69 sites (Figure S2). Within a wide range of pharmacological solutions, the most widely used drugs, in ≥80% of the sites, were: paracetamol, opioids, steroids, non-steroid anti-inflammatory drugs, neuromodulators (neuropeptides, cytokines, etc.), gabapentinoids, antidepressants and metamizole. The use of transdermal capsaicin and intravenous lidocaine was more frequent at public sites, yet statistical significance was only reached for the former after adjustment for multiplicity, whilst hyaluronic acid and platelet-rich plasma injections were more frequent at private sites (statistical significance was not reached in the case of hyaluronic acid). Also most sites offered peripheral nerve blocks, epidural injections, nerve-root (trunk) and intra-articular blocks, radiofrequency and sympathetic blocks. Psychological interventions were available in less than 50% of the sites (Figure S2). In many sites (12/16, 75.0%, detailed data not provided) the physicians liked to enhance their practices by incorporating transcutaneous electrical nerve stimulation, and in more than one-half (10/18‑14/22, 55.6‑63.6%) by including psychotherapy, intrathecal pump implants, implantable devices, and iontophoresis.

Figure S2: Available therapeutic procedures at surveyed sites. Multiplicity adjusted p-values (from Chi-square or Fisher’s exact test) are indicated when there is a statistically significant difference between the groups (public and private). NSAIDs: Non-steroid anti-inflammatory drugs, TENS: Transcutaneous electrical nerve stimulation.

# References

1. C. Barutell, "[Pain units in Spain: Survey of the Spanish Society of Pain, Pain Day 2007]," *Revista de la Sociedad Española del Dolor*, vol. 16, pp. 421-428, 2009.

2. J. R. Gonzalez-Escalada, A. Camba and I. Sanchez, "[Census Pain Units in Spain: analysis of organizational structure, staffing, portfolio services and quality indicators]," *Revista de la Sociedad Española del Dolor*, vol. 21, pp. 149-161, 2014.

3. M. J. Martinez Zapata, E. Catala and M. Rigola, "[Descriptive analysis of chronic pain clinics operating in Spain in 2001]," *Revista Española de Anestesiología y Reanimación*, vol. 52, no. 3, pp. 141-148, 2005.

4. M. Polo-Santos, S. Videla-Ces, C. Perez-Hernandez et al., "An Update on Resources, Procedures and Healthcare Provision in Pain Units: A Survey of Spanish Practitioners," *International Journal of Environmental Research and Public Health*, vol. 18, no. 2, 2021.

5. Ministry of Health Social Welfare and Consummer Affairs, "Framework Document to Improve the Management of Pain in the National Health System," Ministry of Health Social Welfare and Consummer Affairs, Madrid, Spain, 2014.

6. I. Palanca, M. M. Puig, J. Elola et al., "Pain Units: Quality and Safety Standards and Recommendations," Technical Secretary, Ministry of Health, Social Policy and Parity, Madrid, Spain, 2011.

7. P. A. Harris, R. Taylor, R. Thielke et al., "Research electronic data capture (REDCap)--a metadata-driven methodology and workflow process for providing translational research informatics support," *Journal of Biomedical Informatics*, vol. 42, no. 2, pp. 377-381, 2009.

8. A. A. Joyce, A. Conger, Z. L. McCormick et al., "Changes in Interventional Pain Physician Decision-Making, Practice Patterns, and Mental Health During the Early Phase of the SARS-CoV-2 Global Pandemic," *Pain Medicine*, vol. 21, no. 12, pp. 3585-3595, 2020.

# Tables

## Table S1 Description of the survey questionnaire

| **General questions, respondents’ background and participation in teaching/research activities** |
| --- |
| - Date of birth |
| - Gender (male/female) |
| - Medicine Degree (year) |
| - Specialization (year) |
| - Medical specialty (from a list of options) |
| - Experience (years), type of practice (public/private, size), teamwork (yes/no), affiliation to a pain unit (yes/no) |
| - Types of pain treated (acute, chronic, nociceptive, neuropathic, mixed) |
| - Treatment of oncologic pain (yes/no) |
| - Participation in pain management (hours per week) |
| - Participation in congresses, research studies and contributions to journals (yes/no, number) |
| - Training of rotating physicians (yes/no, specialty) |
| - Participation in training activities (yes/no, type) |
| **Workflow, caseload and medical activity** |
| - Average caseload (patients per year) |
| - Interventional procedures (number per year) |
| - Assessment of patients’ expectations (cure, partial/complete relief, no expectations) |
| - Triage/prioritization of initial visits (yes/no) |
| - Average wait times (no. of days for initial/follow-up visits of cancer/non-cancer patients) |
| - Activities during initial visits (medical history, physical examination, additional investigations, diagnosis, therapeutic plan, etc.) |
| - Information to patients/reports (yes/no, when) |
| - Content of clinical reports (reason for visit, medical history, physical examination, diagnostic tests, diagnosis, treatment) |
| - Process management (yes/no) |
| - Pain management bases (evidences, guidelines, personal expertise, protocols, lectures or conferences) |
| - Medical decision information/sharing with patients (informed consent, recommendations, contact details, surveys) |
| - Evaluation of patients’ medical evolution/trajectories (yes/no) |
| - Appraisal of circumstances, resources, processes, etc. most valued by patients (open question) |
| - Positive and negative issues that influence medical activity (open question) |
| **Organization and management** |
| - Functional and organizational structure (individual practice, hospital department, hospital director, etc.) |
| - ISO certification (yes/no) |
| - Formal setups with other colleagues/departments (clinic sessions, liaison services, criteria for referral) |
| - Sharing of information with referring physicians/institutions (yes/no) |
| - Factors that facilitate/hinder medical activity and patient-physician relationship (kindness and attention, visual contact, equipment and facilities, responsiveness, professionalism, wait times, other) |
| **Resources, equipment and staff** |
| - Availability of a dedicated space (yes/no) |
| - Suitability of assigned spaces (adequate/not adequate) |
| - Approximate size of assigned spaces (in square meters) |
| - Available resources (waiting room, administrative area, consultation desks, rooms for clinic sessions, documentary archives, daycare hospital, dedicated or shared operating theatre, inpatient beds, nursing staff, psychologists, etc.) |
| - Available equipment (direct telephone/internet access, library databases, computerized medical records, basic patient monitoring, resuscitation equipment, instrumentation for nerve blocks, radiofrequency devices, ventilator, ultrasound imaging, etc.) |
| - Resources and equipment wanted/required to improve practice (same list as above) |
| - Expendable/unnecessary resources (same list as above) |
| - Availability of other/allied healthcare professionals (other physicians, nurses, psychologists, physical therapists, etc.) |
| - Staff wanted/required to improve practice (same list as above) |
| - Suitability of available staff (enough/insufficient) |
| **Therapeutic/service range** |
| - Referral patterns (patient’s initiative, primary care, trauma surgeon, neurosurgeon, neurologist, etc.) |
| - Reasons for initial visits (need for interdisciplinary treatment, absence of satisfactory diagnosis, poor pain control, drug overuse/addiction, toxicity/drug adverse reactions, etc.) |
| - Available pharmacotherapy (paracetamol, NSAIDs, opioids, gabapentinoids, antidepressants, ketamine, lidocaine, capsaicin, hypnotics, etc.) and techniques/procedures (joint blocks, epidural blocks, peripheral nerve blocks, sympathetic blocks, intrathecal implants, spinal neuromodulation, iontoforesis, TENS, radiofrequency, ablative neurosurgical techniques, etc.) |
| - Techniques/procedures wanted/required to improve practice (same list as above) |

NSAIDs: Non-steroid anti-inflammatory drugs, TENS: Transcutaneous electrical nerve stimulation.
